# *BRAF* and *NRAS* prognostic values in conjunctival melanoma: analysis and literature review

**DOI:** 10.5935/0004-2749.20230071

**Published:** 2023

**Authors:** Francisco Javier Valentín-Bravo, Álvaro Pérez-Rodríguez, Ciro García-Álvarez, Elena García-Lagarto, María Antonia Saornil-Álvarez

**Affiliations:** 1 Department of Ophthalmology, University Clinical Hospital of Valladolid, Spain.; 2 Department of Anatomic Pathology, University Hospital of Araba, Vitoria-Gasteiz, Álava, Spain.; 3 Department of Anatomic Pathology, University Clinical Hospital of Valladolid, Spain.

**Keywords:** Conjunctival neoplasms, Melanoma, Biomarkers, tumor, Proto-oncogene proteins B-raf, Genes ras, Neoplasias da túnica conjuntiva, Melanoma, Biomarcadores tumorais, Proteínas proto-oncogênicas B-raf, Genes ras

## Abstract

**Purpose:**

Conjunctival melanoma is a rare and aggressive tumor with a propensity for regional and distant metastases. This study aimed to analyze *BRAF/NRAS* markers in conjunctival melanoma and their relationship with tumor recurrences and patient prognosis.

**Methods:**

This retrospective, observational, single-center study included consecutive patients with an anatomopathological diagnosis of conjunctival melanoma, registered between January 1992 and December 2019. *BRAF/NRAS* mutations were analyzed using cobas^®^4800 kit (Roche^®^) in samples obtained by excisional or map biopsy. Additionally, the presence of other associated precancerous or tumor lesions was assessed.

**Results:**

A total of 12 patients with positive histological samples for conjunctival melanoma were included (7 women, 5 men), with a mean age at diagnosis of 60 years and a mean evolution time of 6.38 ± 3.4 years. *BRAF V600E* mutation was observed in three biopsies (25%), similar to *NRAS Q61X* (25%). Recurrences occurred in all patients with positive *BRAF* or *NRAS* mutation, and five of these patients developed systemic dissemination (83.33%). Moreover, four of six patients with mutated *BRAF* or *NRAS* (66.66%) had histopathological findings of tumor or precancerous lesions.

**Conclusions:**

*BRAF* and *NRAS* mutations may be risk factors for recurrence and shorter survival in conjunctival melanoma, which would make these patients candidates for targeted therapies and comprehensive and individualized follow-up. All these data warrant standardized prospective studies.

## Abstract (pt)

**Objetivo:**

O melanoma da conjuntiva é um tumor raro e agressivo, com propensão à disseminação metastática regional e distante. Este estudo tem como objetivo analisar os marcadores *BRAF* e *NRAS* no melanoma da conjuntiva e sua relação com recidivas tumorais e com o prognóstico do paciente.

**Métodos:**

Este foi um estudo retrospectivo, observacional e unicêntrico de pacientes consecutivos com diagnóstico anatomopatológico de melanoma da conjuntiva feito entre janeiro de 1992 e dezembro de 2019. As mutações *BRAF* e *NRAS* foram analisadas com o kit cobas^®^ 4800 (Roche^®^) em amostras obtidas através de biópsia excisional ou por mapa. Além disso, foi avaliada a presença de lesões pré-cancerosas ou tumorais associadas.

**Resultados:**

Foram incluídos 12 pacientes com amostras histológicas positivas para melanoma da conjuntiva (7 mulheres e 5 homens), com idade média ao diagnóstico de 60 anos e tempo médio de evolução de 6,38 ± 3,4 anos. A mutação *BRAF V600E* foi encontrada em 3 biópsias (25%), bem como a *NRAS Q61X* (25%). Ocorreram recidivas em todos os pacientes positivos para mutações de *BRAF* ou *NRAS* e 5 desses pacientes desenvolveram disseminação sistêmica (83,33%). Além disso, 4 dos 6 pacientes com *BRAF* ou *NRAS* mutante (66,66%) apresentaram achados histopatológicos de lesões tumorais ou pré-cancerosas. **Conclusões:** As mutações *BRAF* e *NRAS* podem ser fatores de risco para recorrência e menor sobrevida no melanoma da conjuntiva, o que tornaria esses pacientes candidatos a terapias direcionadas e a um acompanhamento mais abrangente e individualizado. Todos esses dados justificam mais estudos prospectivos padronizados.

## INTRODUCTION

Conjunctival melanoma (CM) is a rare and aggressive malignant neoplasm originating on the ocular surface of melanocytes. They represent 1%-2% of ocular malignancies and generally affect Caucasian adults without gender preference^(1,2)^. Despite low CM incidence, estimated at 0.2-0.8 cases per million inhabitants/year in Caucasians, it is a potentially life-threatening cancer with a 10-year mortality of 23%-30%^(2-4)^. Additionally, it typically presents as a raised mass of variable pigmentation, which, in up to 15%-19% of cases, may be amelanotic^(5,6)^. Although it may originate *de novo* in 5%-10% of patients, the association between CM and two related conditions, such as primary acquired melanosis (PAM) in 75% and conjunctival nevus in 20%-30%, has been well established^(2)^.

The main predictive factor for metastasis is the depth of tumor invasion, and metastasis is very unlikely if the thickness of the tumor is less than 0.8 mm^(7)^. Other low prognostic factors described are tarsal or caruncle location, a high proliferation index (Ki67), pagetoid histological pattern, or recurrence^(8)^. Local recurrence is frequent, and if the disease disseminates, the survival rate decreases markedly. However, there is no approved standard treatment for metastatic CM^(6)^. Therefore, a better understanding of the genetic CM profile may help identify therapeutic targets for patients with advanced disease stages.

Although CMs have not been well-characterized genetically, cutaneous melanomas share specific chromosomal alterations with them and frequently harbor pathogenic variants in *BRAF* (50%), *NRAS* (20%), or *NF1*^(9,10)^.

Pro-oncogenic *BRAF* mutations are present in 14%-50% of CM cases, with *BRAF V600E* being the most frequent (74%-82%), followed by *V600K* (12%-20%). Although other rare *BRAF* mutations can be found and account for 6%, the determination of those rare mutations is not cost effective in clinical practice^(10)^. Additionally, *NRAS* mutations have been detected in up to 18% of the analyzed samples, and other mutations, such as telomerase reverse transcriptase promoter and *c-KIT* mutations, occur in 32%-41% of CM cases. These mutations were thought to be mutually exclusive in the past; however, studies have shown that there may be a coexistence between these mutations^(11,12)^.

A better understanding of the genetics and molecular abnormalities involved in CM progression would allow the identification of the most locally and systemically aggressive lesions and improve these patients’ survival by amplifying existing therapies use and developing new therapeutic targets.

This study aimed to analyze *BRAF/NRAS* markers in CM and consider their relationship with tumor recurrences and patients’ prognosis.

## METHODS

This was a retrospective, observational, and single-center study, including consecutive patients registered in Ocular Tumor Unit Hospital database with a histopathological diagnosis of CM from January 1992 to December 2019. The selection process included patients with confirmed diagnoses in other hospital centers referred for treatment and initial pathology recurrences. Additionally, samples were collected from patients with melanoma who had undergone excisional or map biopsy.

**Figure 1:**
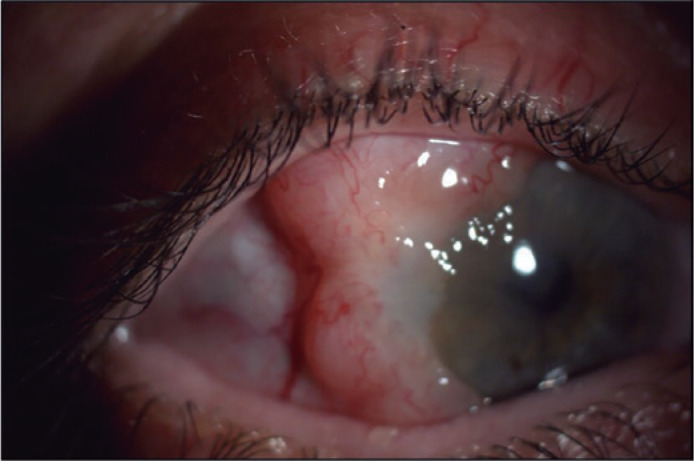
Clinical image of amelanotic conjunctival melanoma.

Patients’ clinical history was reviewed, paying particular attention to information related to demographics, ophthalmologic examination (Figure 1), biopsy type, histopathological diagnosis, recurrences, and presence of regional or systemic metastasis.

**Figure 2:**
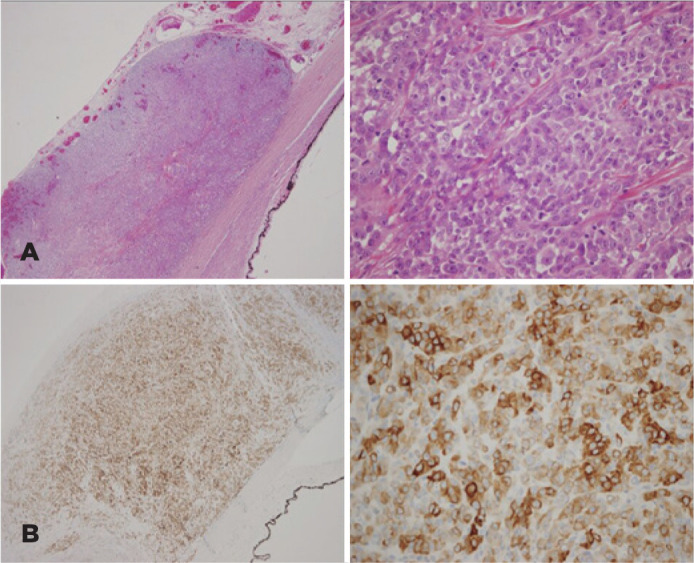
(A) Hematoxylin and eosin ×4/×40: The tumor consisted of epithelioid cells with atypical nuclei and eosinophilic cytoplasm. A neoplastic proliferation of melanocytic lineage consisting of epithelioid cells with atypical nuclei and multiple mitotic figures is observed. (B) Melan A ×4/×40: The tumor cells showed intense and diffuse cytoplasmic expression for Melan A, confirming its melanic origin.

The histological diagnoses based on hematoxylin-eosin and the immunohistochemical staining performed (*S100, SOX10, MELAN A, and Ki67*) were reviewed by Anatomic Pathology service, adding new sections when necessary and the amount of available tissue allowed it (Figure 2). *BRAF/NRAS* mutation analysis was performed using cobas^®^4800 kit (Roche^®^). *BRAF V600* mutation test using cobas^®^ 4800 detects *BRAF V600* mutations in DNA extracted from formalin-fixed, paraffin-impregnated human melanoma tissue. It is a real-time polymerase chain reaction (PCR) assay designed to detect *V600E* mutation. Cobas^®^ 4800 *BRAF V600* mutation test is based on the following two processes: (1) manual sample preparation to obtain genomic DNA from formalin-fixed, paraffin-embedded tissue and (2) target DNA PCR amplification and detection using a pair of complementary primers and two oligonucleotide probes labeled with different fluorescent dyes. One search is aimed to detect wild-type *BRAF V600*, and another is aimed to detect *V600E* mutation sequence. Two external run controls were provided, and the wild-type allele served as internal complete process control. For this, a 5-µm deparaffinized section of a paraffin-fixed sample was lysed by incubation at elevated temperature with a protease and lysis/chaotropic binding buffer that releases nucleic acids and protects the released genomic DNA from DNAases. Subsequently, isopropanol was added to the lysis mixture and centrifuged through a column containing a glass fiber filter. During centrifugation, genomic DNA binds to the glass fiber filter surface. Unbound substances, such as salts, proteins, and other cellular impurities, are removed by centrifugation. Genomic DNA amount was determined spectrophotometrically and adjusted to a fixed concentration and the amplification and detection mixture. The target DNA was then amplified and detected using the amplification and detection reagents included in the cobas^®^ 4800 *BRAF V600* Mutation Test Kit.

The Clinical Research Ethics Committee approved the present study protocol of Valladolid Eastern Health Area, Hospital Clínico Universitário de Valladolid (CEIC-VA-ESTE-HCUV). This study was conducted in accor­dance with the guidelines of the Helsinki declaration.

## RESULTS

A total of 24 histological samples with CM anatomopathological diagnosis was included. However, to detect *BRAF* or *NRAS* mutations, PCR was only performed in 12 biopsies. This study could not be performed in the remaining 12 samples due to insufficient material for DNA extraction or because they were not available in our center.

The details of the 12 included patients are shown in table 1. Sex distribution was almost similar, with seven females (58.33%) and five males (41.67%). The mean subjects’ age at diagnosis was 60 years (median 58; range 35-88 years), with a slightly lower age at presentation in patients without the mutation, 57.16 vs. 62.83 years with positive mutation. The right eye (RE) was predominantly affected (10 occasions; 83.33%). A total of 10 tumors presented multicentrically (83.33%), and the remaining 2 had a circumscribed presentation (16.67%). Considering melanomas origin, nine developed from PAM (75%) and one developed from melanocytic nevus malignization (8.33%). The remaining two CMs developed *de novo* (16.67%) without a previous predisposing lesion. The mean follow-up was 6.38 ± 3.4 years.

**Table 1:** General subjects’ characteristics

Sexo	Edad	Tumor shape	Origin	Recurrences	Systemic	Death	BRAF V600E	NRAS (Q61X)	Other tumors
F	58	Multicenter	PAM	4	No	No	No	No	No
F	57	Circumscribed	PAM	1	Yes	No	No	Yes	Adenoma villous sigma
M	82	Multicenter	PAM	1	Yes	Yes	Yes	No	Intestinal polyps. pulmonary hamartoma. cutaneous melanoma
F	47	Multicenter	PAM	No	No	No	No	No	No
F	35	Multicenter	PAM	2	Yes	No	No	No	Renal angiomyolipoma
F	70	Multicenter	PAM	4	Yes	Yes	No	Yes	No
F	88	Multicenter	PAM	No	No	No	No	No	No
F	40	Circumscribed	Nevi	4	Yes	Yes	No	Yes	No
M	55	Multicenter	PAM	No	No	No	No	No	No
M	60	Diffuse	Melanoma	No	No	No	No	No	No
M	70	Multicenter	Melanoma	2	Yes	No	Yes	No	Cutaneous squamous cell carcinoma/keratoacanthoma
F	58	Multicenter	PAM	1	No	No	Yes	No	Gastric fundus and cecum polyp. xanthoma. uterine myoma

M= male; F= female; PAM= melanosis adquirida primaria.

*BRAF* V600E mutation was observed in three patients with CM, accounting for 25% of cases. Additionally, three other biopsies were positive for Q61X mutation in *NRAS*.

Recurrence was observed in all patients with positive *BRAF* or *NRAS* mutation, whereas only two of the six patients without the mutations developed recurrence. Likewise, metastases were identified in 5 *BRAF*- or *NRAS*-positive samples, of which three died. However, only one of the six mutation-negative patients had locoregional or systemic involvement (16.67%).

More aggressive treatment with enucleation or exenteration was performed in three patients, all of whom had a previous recurrence and two of whom possessed *NRAS* mutations.

Of the 12 patients, 5 had been diagnosed with pre­cancerous or cancerous lesions in other locations. Specifically, four of the six patients with mutated *BRAF* or *NRAS* (66.66%) had histopathological findings of such lesions as cutaneous melanoma, pulmonary hamartoma, uterine myoma, or intestinal and gastric polyps. On the contrary, such a lesion was only found in one patient out of the total without mutation.

## DISCUSSION

CM is an uncommon pathology with high mortality due to potential metastasis and recurrence^(13)^. Despite the absence of strict clinical practice guidelines for disease management, every patient suspected of this neoplasm should be directed to a referral center^(6)^. On the one hand, local adjuvant treatment has failed to improve survival in CM patients, especially in those with metastatic disease; thus, the search for targets and new therapeutic options is necessary^(14)^. Recent publications have proposed targeted therapy against CM with *BRAF/NRAS* mutations, possibly improving patient prognosis^(15)^.

The present study results support the hypothesis of the relationship between recurrence and worse prognosis in CM patients with mutated *BRAF* and *NRAS*^(16)^. This fact makes these patients subsidiary to the abovementioned therapies.

Despite the small sample size included in the study (n=12), few studies have related the described mutations with disease prognosis and their association with other tumors.

These findings should be evaluated with caution due to certain limitations, such as its retrospective design and the referral bias inherent in an ocular ophthalmology referral unit. Similarly, a small patients’ number due to low disease prevalence and the limited sample size for the histological study constitute a barrier to the statistical research. Another limitation comprises the absent analysis of different *BRAF* variations, such as *V600K,* or alterations in *NF1*, present in up to 14% of tumors^(17)^, resulting in underestimation of the mutations in the sample.

In this study, we present the clinical and genetic data of 12 patients with a histopathological diagnosis of CM. No differences were found in terms of sex or age at presentation. However, CM with alterations in these markers has been occurring more frequently in young males^(18,19)^, although without statistical significance.

The study showed positivity for *BRAF V600E* and *Q61X NRAS* mutations in 25% of the cases each, which is similar to the data described by Griewank et al.^(20)^ (29% and 20%). However, other publications, such as Kenawy et al.^(18)^ and Larsen et al.^(19)^, identified *BRAF V600E* mutations in primary tumors with much higher numbers, 40.4% and 50%, respectively. Recently, Gkiala et al.^(17)^ have shown that in a literature review with 563 CMs, 29.7% (n=167) harbored *BRAF* mutations and only 6.4% (n=36) harbored *NRAS* mutations. In our study, these differences may be explained by the insufficient material amount in the samples, the detection method, or the absence of searching for other infrequent mutations in *BRAF*, such as *V600K* or *T1799A*^(16,21)^.

Although recent studies have correlated *BRAF/NRAS* mutations with advanced CM^(16)^, the influence of these biomarkers on disease prognosis is still uncertain^(22)^. However, their identification provides a possibility of a targeted systemic chemotherapeutic treatment that could increase patients’ survival with disseminated disease^(3)^. Some authors reported that mutated CMs show a higher metastatic tendency and lower survival^(16,23,24)^, whereas others did not confirm this correlation^(25)^. In our study, disease recurrence was documented in all patients with *BRAF* or *NRAS* mutations, and 83.3% of them had disseminated disease during the follow-up. Additionally, 50% of patients died from the primary tumor. This suggests that mutation implies a higher risk of recurrences and metastases, worsening the prognosis for life.

Since 2002, *BRAF* and *NRAS* mutations have been described in a wide range of benign neoplasms and up to 8% of malignancies^(26)^. These include cutaneous melanoma, colon adenocarcinoma (10%-15%), gastrointestinal polyps^(27)^, thyroid carcinoma (45% papillary), and metanephric adenoma^(28)^. Despite the high prevalence of gastrointestinal polyps in the general population, these precancerous lesions were observed on biopsy in three of the six patients with mutations. In addition, cutaneous melanoma was found in a BRAF-positive patient. This would support studies, such as Rabbie et al. 2019, which concluded that *BRAF* gene mutation acts as a driver mutation in the early stage of tumorigenesis^(29)^.

Finally, recent publications, such as Zeng et al.^(30)^, have recommended the routine detection of *BRAF* mutations in patients with advanced CM to individualize their treatment and follow-up.

In conclusion, *BRAF* and *NRAS* mutations may be risk factors for disease recurrence and shorter survival in CM, warranting targeted therapies and a more detailed and individualized follow-up. However, future prospective multicenter studies that include a larger sample size are needed to confirm the predictive value of these mutations and evaluate the efficacy and safety of drugs aimed at these therapeutic targets.
